# Infected deep vein thrombophlebitis in people who inject drugs: missed opportunities and potential for alternative antimicrobial approaches

**DOI:** 10.1007/s15010-021-01725-3

**Published:** 2021-11-02

**Authors:** Hugh McCaughan, Clark D. Russell, Dáire T. O’Shea

**Affiliations:** 1 Clinical Infection Research Group, Regional Infectious Diseases Unit, Western General Hospital, Edinburgh, UK; 2 Centre for Synthetic and Systems Biology, Waddington Building, Kings Buildings, Edinburgh, UK; 3 University of Edinburgh Centre for Inflammation Research, Queen’s Medical Research Institute, Edinburgh, UK

**Keywords:** Opioid use disorder, Substance use disorders, Deep vein thrombosis, Thrombophlebitis

## Abstract

Infected deep vein thrombophlebitis (i-DVT) in people who inject drugs (PWID) is a clinically challenging but poorly characterised disease. We undertook a retrospective observational study of 70 PWID presenting acutely with i-DVT to improve the clinical and microbiological characterisation of this disease. i-DVT was frequently associated with bacteraemia (59.1% patients with blood cultures obtained), groin abscesses (in 34.3%; of which 54.2% required surgical drainage), and septic pulmonary emboli (38.6%) requiring anticoagulation. Network analysis identified a cluster of patients presenting with respiratory symptoms but lacking typical DVT symptoms, more likely to have septic pulmonary emboli. A microbiologic diagnosis was frequently achieved (70%). Causative pathogens were predominantly gram-positive (*S. aureus* and streptococci, especially anginosus group), whereas gram-negative pathogens were identified very infrequently (in 6.1% of microbiological diagnoses). This suggests routine empiric therapy against gram-negative bacteria, though commonly administered, is not required. High rates of clinical cure (88.6%) were observed despite the complex nature of infections and independently of the highly variable intravenous and total antimicrobial durations received. There exists a rationale to devise pragmatic approaches to implement novel individualised treatment plans utilising oral antimicrobial therapy for i-DVT. Despite frequent healthcare interactions, opportunities to address HCV treatment and opioid substitution therapy were frequently missed during these acute admissions.

**Supplementary Information:**

The online version contains supplementary material available at 10.1007/s15010-021-01725-3.

## Introduction

People who inject drugs (PWID) are at increased risk of bacterial disease complicating injections, including cellulitis, abscesses and infective endocarditis (IE). Infected deep vein thrombophlebitis (i-DVT) is estimated to account for up to 11% of infectious complications amongst hospitalised PWID, based on a single-centre Swiss retrospective observational study [1]. I-DVT is a clinically challenging entity but poorly characterised. Optimal management is not defined, particularly in comparison to IE in PWID [1–3]. In general, PWID are a therapeutically disenfranchised group, often excluded from potentially relevant clinical trials, contributing to a lack of data to guide therapeutic decisions. To improve the clinical characterisation of this entity, we undertook a retrospective observational study of PWID presenting acutely with i-DVT.

## Methods

### Case acquisition

Cases were defined as (1) adults currently injecting drugs intravenously, presenting with (2) radiologically confirmed upper or lower limb DVT (excluding isolated superficial thrombophlebitis) with (3) evidence of infection, either (i) radiological or intra-operative (inflammatory vessel changes associated with thrombus, gas in thrombus, vessel with thrombus contiguous with abscess) or (ii) microbiological (bacteraemia or culture-positive intra-operative thrombus sample). Electronic patient records and Infectious Disease consultation records were searched for the terms “DVT”, “PWID” and “IVDU” for patients admitted to infectious disease, vascular surgery or critical care wards from January 2017–December 2018 inclusive, identifying 2957 acute admissions, 70 of which met the case definition and had available records. This work was approved by the NHS Lothian Infection Service Quality Improvement Team.

### Analysis

Microbiological cure was defined as a negative blood culture (BC) following a positive BC. Clinical cure was defined as no re-admission due to the same infection within one year of discharge. Symptom clusters were identified by network analysis using the Markov Clustering Algorithm (Graphia, version 2.0 [4]). Groups were compared using unpaired t-tests, Mann–Whitney tests or Fisher’s exact tests as appropriate.

## Results

### Cohort characteristics

Patients were predominantly male (46/70; 65.7%) with a median age of 37 years (Table 1, Table S1). Prior to admission 10/70 (14.3%) patients had no fixed abode and 10/70 (14.3%) resided in non-permanent accommodation. All patients injected heroin with 41/70 (58.6%) using additional substances, most frequently cocaine and/or benzodiazepines (Table 1; Figure S1). Active hepatitis C was present in 22/70 (31.4%), with 7 of these being new diagnoses during the index hospitalisation. Thirty-six patients (51.4%) had previously been hospitalised due to complications of injection drug use (median of 1, IQR 1–3, range 1–15), commencing a median of 33 months (IQR 20–64.75) previously, commonly due to cellulitis (*n* = 28) and/or deep vein thrombosis/pulmonary embolism (DVT/PE; *n* = 19).

**Table 1:** Cohort characteristics

Variable	*N* (%) (*n* = 70)^a^
Demographics	
Male:Female	46:24
Age, median (IQR) years	37 (32–41)
Temporary accommodation or no fixed abode	20 (28.6)
Substance use	
Alcohol excess	10 (14.3)
Heroin only	29 (41.4)
+ Cocaine	26 (37.1)
+ Benzodiazepines	26 (37.1)
+ Cannabis	9 (12.9)
+ New psychoactive substance	5 (7.1)
+ Others	6 (8.6)
Blood borne viruses	
HIV positive	0
Active HBV	2 (2.9)
Active HCV	22 (31.4)
Prior cleared HCV	15 (21.4)
Presentation	
Symptom duration prior to admission, median (IQR) days	4.5 (3–7)
Vital signs, median (IQR)	
Pulse, beats/min	102 (85–118)
Systolic BP, mmHg	113 (90–123)
SpO_2_, %	97 (95–99)
Temperature, °C	38.4 (37.5–39)
Respiratory rate, breaths/min	17 (16–19)
Antimicrobial durations, median (IQR) days^b^	
Intravenous	18 (7–29)
Oral follow-on (*n* = 54)	14 (14–27)
Combined	29 (28–43)
Surgical intervention	
Abscess drainage	13 (18.6)
Pseudoaneurysm ligation	4 (5.7)
Complications	
Bacteraemia	39/66 (59.1)^c^
Septic pulmonary emboli	27 (38.6)
Groin abscess	24 (34.3)
Arterial involvement	8 (11.4)
Pseudoaneurysm	6 (8.6)
Splenic emboli	3 (4.3)
Infective endocarditis	3 (4.3)
Outcomes	
Clinical cure	62 (88.6)
Microbiological cure	38/39 (97.4)
Substance-use related admission in year after discharge	32 (45.7)

Data shown as *n (%)* unless otherwise stated

^a^Denominator = 70 unless otherwise stated

^b^Data available for *n* = 68

^c^Blood cultures obtained in *n* = 66

*IQR* interquartile range, *HIV* human immunodeficiency virus, *HBV* hepatitis B virus, *HCV* hepatitis C virus, *BP* blood pressure, *SpO*_*2*_ oxygen saturations

### Presenting features

Network analysis identified distinct symptom clusters. Combinations of leg pain with/without swelling and fever were the most common presenting symptoms, but one cluster of patients presented predominantly with fever and respiratory symptoms (≥ 1 of cough, dyspnoea or pleuritic chest pain; Table S1, Figure S2). Laboratory evidence of systemic inflammation was common (median CRP 220 mg/L [IQR 175–292]; median neutrophils 9.8 × 10^9^/L [6.9–13.9]) but overt sepsis (qSOFA ≥2) was uncommon (7/60; 11.7%) (Table 1, Figure S3). Thirteen patients were admitted to the intensive care unit. Radiologic confirmation of DVT was by US (46/49; 93.9%) and/or CT (36/55; 65.6%), identifying thrombi most commonly in the common femoral (*n* = 29), external iliac (*n* = 17) or common iliac (*n* = 14) veins. Abnormalities were present in 21/61 (34.4%) chest x-rays (cavitating pneumonia in four). Thoracic CT (pulmonary angiogram 24; venous contrast 13) identified definite emboli in 6/37 (16.2%) and cavitation in 23/37 (62.2%) patients who underwent imaging; more often in the respiratory symptom cluster.

### Microbiological findings

Bacteraemia was identified in 39/66 (59.1%) patients with BC obtained at presentation (9 polymicrobial). Bacteraemic patients had a median of 1 positive BC per organism (range 1–3). Seven patients had > 1 positive BC; in all cases *Staphylococcus aureus*. Intra-operative samples were culture-positive from 16/19 (84.2%) patients where obtained. Considering BC and intra-operative samples together, a microbiological diagnosis was made in 49/70 (70%) patients; monomicrobial in 32/49 (65.3%). Most monomicrobial infections were due to *S. aureu*s (21/32, 65.6%). *S. aureus*, streptococci (especially anginosus group) and anaerobes were the most commonly identified pathogens overall (Table S2). Gram-negative organisms were infrequently identified (3 patients).

### Antimicrobial therapy

**Fig. 1:**
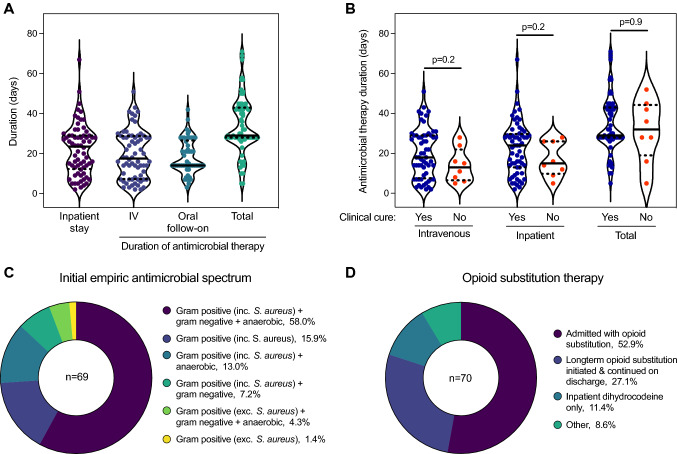
Inpatient management. **A** Duration of inpatient stay and antimicrobial therapy (intravenous [IV], oral, and total [IV and oral combined]). **B** Comparison of the duration of intravenous, inpatient and total antimicrobial therapy between patients with and without clinical cure. Data available for 68 patients. Groups compared by Mann Whitney test. Solid line within violin plot shows median and dotted lines show first and third quartiles. **C** Initial spectrum of empiric antimicrobial therapy. Definitions of different spectra are presented in Supplementary Table 3. **D** Opioid substitution therapy

All patients (*n* = 68 data available) were initially treated with intravenous antimicrobials continuing for a median of 18 days (IQR 7–29), with oral follow-on therapy in 54/68 (79.4%) patients, prescribed for a median of 14 days (IQR 14–27), resulting in a median total duration of 29 days (IQR 28–43) but with substantial variation (Fig. 1A). The median inpatient stay was 24 days (IQR 12–29). Adherence to oral antimicrobial therapy after discharge is unknown, thus these data are the upper bound to oral and total durations. An enforced early IV-to-oral switch occurred in three patients due to intravenous access difficulties and in eight due to discharge against medical advice.

Initial empiric antimicrobial therapy favoured *S. aureus* coverage, with 65/69 (94.2%) patients receiving agents with activity against gram-positive pathogens including *S. aureus* (Fig. 1C; Table S3). In 40/69 (58%) patients, initial empiric therapy also included gram-negative and anaerobic coverage. Total antimicrobial days were summed and the proportion accounted for by each ‘spectrum’ was derived (Table S4). Specific anti-staphylococcal gram-positive therapy accounted for 42.4% of antimicrobial days, followed by anaerobic therapy (24.9%), broad-spectrum therapy (13.0%), narrow-spectrum gram-positive therapy (9.9%) and gram-negative therapy (8.8%).

### Other management

Peripherally inserted central catheters or midlines, inserted by an anaesthetist or radiologist (excluding central venous access for vasopressors), were required for intravenous access in 32/70 (45.7%) patients. Surgical intervention was required in 22/70 (31.4%) patients, most commonly for abscess drainage (*n* = 13) or pseudoaneurysm ligation (*n* = 4). Therapeutic anticoagulation was initiated with subcutaneous low molecular weight heparin (LMWH) for most patients (57/70; 81.4%) or LMWH followed by a direct-acting oral anticoagulant (DOAC; n = 7) or DOAC alone (*n* = 1). Four patients were not anticoagulated and one was already taking therapeutic anticoagulation. The majority of patients received long-term opioid substitution therapy, however, 14/70 (20%) patients were discharged without a treatment plan (Fig. 1D). Sixteen (of 22; 72.7%) patients with active HCV infection were offered outpatient treatment; only six ultimately received treatment. In five patients active HCV infection was not addressed. Data was unavailable for one patient with active HCV.

### Complications

Septic pulmonary emboli (*n* = 27 [38.6%]; either definite emboli or cavitation) and groin abscesses (*n* = 24, [34.3%]; of which 13 underwent drainage [54.2%]) were the most common complications. Adjacent arterial involvement (*n* = 8; 11.4%) and pseudoaneurysms (*n* = 6; 8.6%) were less common. Other metastatic complications included: splenic emboli (*n* = 3; 4.3%), IE (*n* = 3 [4.3%]; 50/66 underwent echocardiography), vertebral osteomyelitis (*n* = 1; 1.4%), septic arthritis (metatarsophalangeal joint, *n* = 1; 1.4%), pulmonary artery pseudoaneurysm requiring surgical intervention (*n* = 1; 1.4%), right ventricle thrombus containing an embolised needle tip (*n* = 1; 1.4%) and necrotising fasciitis of the groin and abdominal wall (*n* = 1; 1.4%).

### Outcomes

Amongst patients with data available, microbiological cure was achieved in 38/39 (97.4%) cases. Clinical cure was achieved in 62/70 (88.6%) cases and there were no deaths during the index admission. Clinical failure leading to re-admission occurred an average of 17 days after discharge (SD ± 9, range 5–26). 32 (45.7%) patients had a further admission within one year due to other complications of injection drug use. Bacteraemia, *S. aureus* disease, septic shock, qSOFA/SIRS, inflammatory markers, metastatic infection and local abscess were not associated with clinical failure on univariable analyses. Duration of intravenous, inpatient or total antimicrobials was also not associated with outcome (Table S5, Fig. 1B).

## Discussion

i-DVT in PWID was frequently associated with bacteraemia, groin abscesses and septic pulmonary emboli. Patients often received lengthy antimicrobial courses associated with extended hospitalisations. Despite the complex nature of infections, outcomes were surprisingly good, with clinical cure in 88.6% of patients. Moreover, cure occurred independent of highly variable durations of antimicrobial therapy. Patient characteristics and outcomes reported here are similar to other smaller studies (Table S6) [1, 5, 6]. In a North American cohort of PWID with invasive bacterial infections (predominantly IE and bone/joint infections), outcomes were similar for patients who completed a prolonged intravenous antimicrobial course and those who required partial oral therapy (and superior to those who did not complete intravenous therapy nor receive partial oral therapy) [7]. Combined with data supporting partial oral therapy for the treatment of IE and osteomyelitis (in studies excluding PWID [8, 9]), we contend there exists a rationale to devise pragmatic approaches to implement novel individualised treatment plans utilising oral antimicrobial therapy for i-DVT. A microbiologic diagnosis was frequently achieved (49/70 patients). *S. aureus*, streptococci and anaerobes were the most commonly identified pathogens whereas gram-negative pathogens were identified very infrequently (in 3/49 patients with a microbiological diagnosis). Considering the rarity of identifying gram-negative pathogens, we propose that routine use of broad spectrum/gram-negative therapy empirically is not required.

Although treatment failure of the index infection was uncommon, readmissions within one year with other complications of injection drug use occurred in almost half the cohort (45.7%), justifying intensification of efforts to address causation. Fourteen patients (20%) were discharged without addressing opioid substitution therapy. Active HCV was common (31.4%) but only a minority were successfully engaged in treatment. There was minimal documentation regarding harm reduction measures. In Scotland, drug-related acute admissions are increasing steadily with 10,509 admissions in 2017–18 (199 stays/100,000 population), representing 7,986 distinct patients of which 54% had not been admitted before[10].

The generalisability of these results is limited by the single-centre design. Our ability to identify predictors of treatment failure was limited by the low event rate. Patients re-admitted to hospitals outwith the geographic area of the study would not have been identified so the figures presented for clinical cure represent an upper bound (i.e. we may have missed cases of clinical failure); however local experience suggests this is unlikely to have been a frequent event.

This report addresses a deficit in the clinical and microbiological characterisation of i-DVT in PWID, providing a basis for reformed antimicrobial approaches. Despite frequent interactions and prolonged hospital stays, we also identify missed opportunities to address causal factors and more specifically to target the reservoir of active HCV in PWID. PWID presenting acutely with i-DVT represent a high-risk group in need of an integrated, holistic approach to management and efforts to facilitate healthcare engagement.

## Supplementary Information

Below is the link to the electronic supplementary material.

Supplementary file 1 (PDF 2622 KB)

## Data Availability

Anonymised raw data available on reasonable request.
