# Supplementary material for: Infected deep vein thrombophlebitis in people who inject drugs: missed opportunities and potential for alternative antimicrobial approaches
Source: Infection. 2021 Nov 2;50(2):507–11. doi: 10.1007/s15010-021-01725-3 (PMC8942891; doi:10.1007/s15010-021-01725-3)
Supplement: Supplementary file 1 — (PDF 2622 KB) [file 15010_2021_1725_MOESM1_ESM.pdf]

Infected deep vein thrombophlebitis in people who inject drugs: missed opportunities and potential for alternative antimicrobial approaches.

Hugh McCaughan, Clark D. Russell, Dáire T. O'Shea

SUPPLEMENTARY DATA

Contents

Supplementary Figure 1: Combinations of substances used 2

Supplementary Figure 2: Symptom clusters 3

Supplementary Figure 3: Laboratory parameters on admission 4

Supplementary Table 1: Additional cohort characteristics 5

Supplementary Table 2: Microbiological findings 7

Supplementary Table 3: Classification of antimicrobial spectra 7

Supplementary Table 4: Cumulative antimicrobial usage 8

Supplementary Table 5: Univariable analyses of factors associated with clinical failure 9

Supplementary Table 6: Findings from previous observational studies 9

References 10

FIGURES

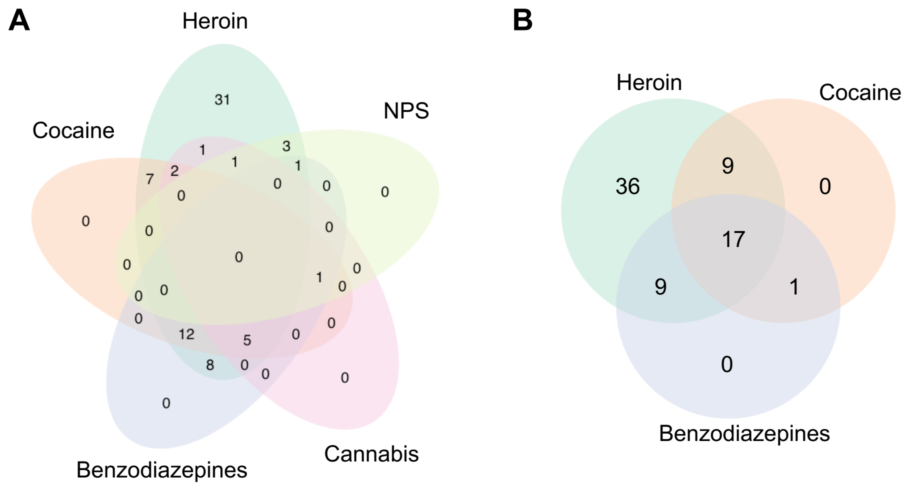

Supplementary Figure 1: Combinations of substances used
(A) Overlap in use of all specified substances. **(B)** Overlap in use of most commonly used substances. NPS: new psychoactive substances.

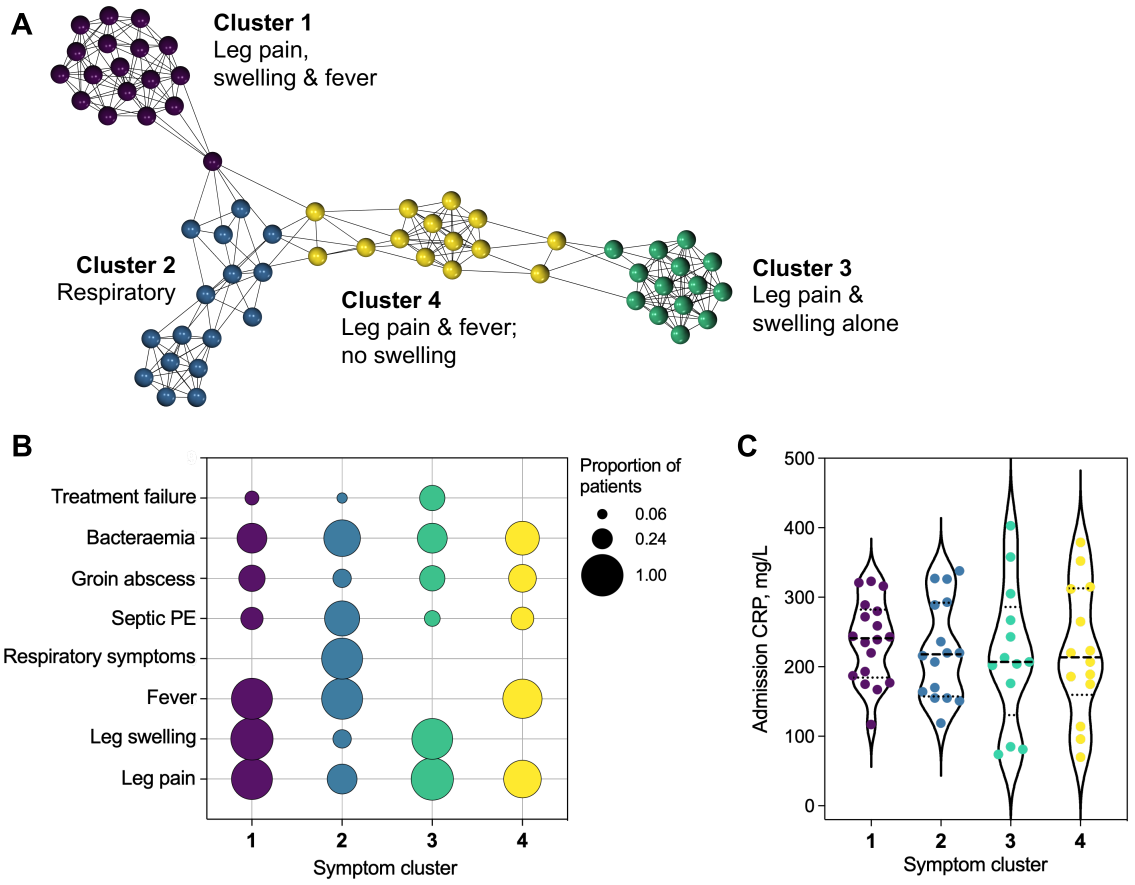

Supplementary Figure 2: Symptom clusters

(A) Patient-to-patient network analysis. Symptoms documented on admission (leg pain, leg swelling, fever and respiratory [≥1 of cough, dyspnoea or pleuritic chest pain]) were used to identify four clusters of patients. Edges represent connections with a Pearson correlation value of ≥0.5 (chosen to keep all patients in the network). k-Nearest-Neighbours method was used for edge reduction (k=5). Nodes represent patients and are coloured by cluster membership, determined using the Markov Clustering Algorithm (a coarse granularity of 1.5 was applied to avoid excessive division of patients into small uninterpretable clusters). Patients with any missing symptom data were excluded leaving n=62. **(B)** Bubble plot representing proportion of patients in each symptom cluster with the listed feature. Size of bubble indicates proportion of patients. Bacteraemia is presented as a proportion of patients who had blood cultures taken. **(C)** C-reactive protein (CRP) concentration at admission did not differ between the symptom clusters. Dashed line within violin plot shows median and thin dotted lines show first and third quartiles.

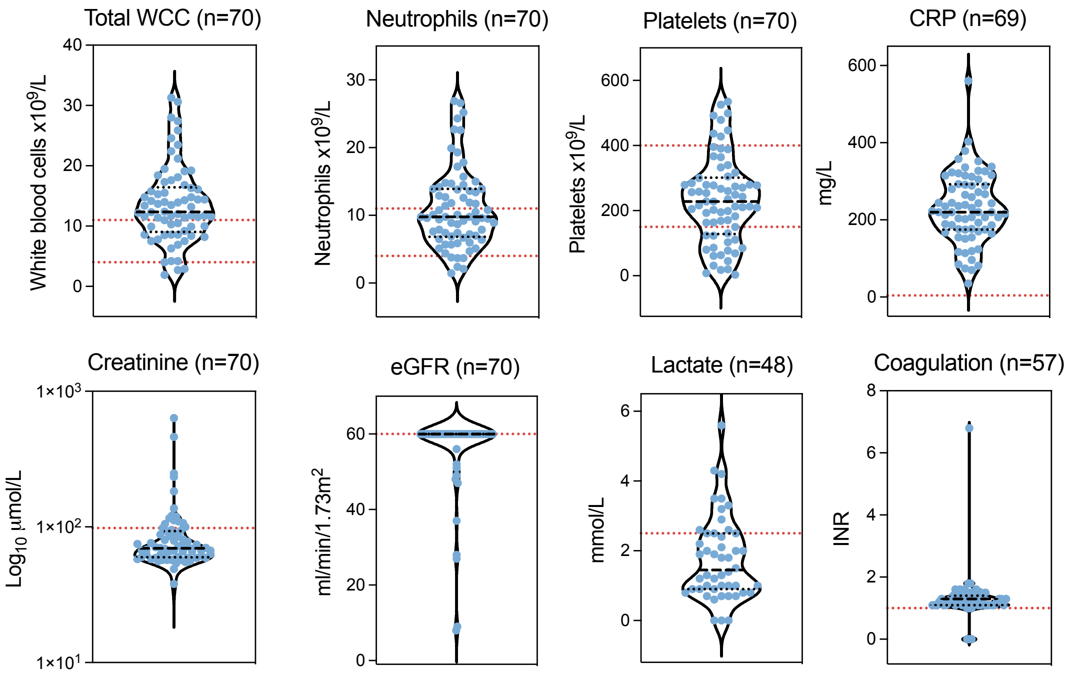

Supplementary Figure 3: Laboratory parameters on admission

Total white cell count, neutrophil count, platelet count, C-reactive protein, creatinine, estimated glomerular filtration rate, lactate and coagulation measured by internationalised normal ratio (INR). Dashed line within violin plot shows median and dotted lines show first and third quartiles. Red dotted lines on y-axis represent upper and lower limits of normality (if two lines) or upper limit of normality (if single line).

TABLES

Supplementary Table 1: Additional cohort characteristics

Variable	N (%) (n=70)[a]
**Demographics**	
Housing	
Permanent	49 (70)
Temporary	10 (14.3)
No fixed abode	10 (14.3)
**Substance use**	
Alcohol	
None	14 (20)
Recommended limits	37 (52.9)
Harmful use	1 (1.4)
Dependence	9 (12.9)
**Blood borne viruses**	
Human immunodeficiency virus	
Positive	0
Negative	60 (85.7)
Not tested	10 (14.3)
Hepatitis B virus	
Positive (active)	2 (2.9)
Negative	59 (84.3)
Not tested	9 (12.9)
Hepatitis C virus	
Active infection	22 (31.4)
Cleared infection	15 (21.4)
Negative	26 (37.1)
Not tested	4 (5.7)
HCV treatment history	
Never treated	14 (20)
Treated: now negative	3 (4.3)
Treated: failed therapy	1 (1.4)
Spontaneous clearance	15 (21.4)
**Presentation**	
Presenting symptoms	
Pain	49 (70)
Leg swelling	34 (48.6)
Fever	47 (67.1)
Fever alone	7 (10)
Respiratory symptoms	19 (27.1)
Physical examination	
Groin sinus	63/68 (92.6)
Local infection	42/68 (61.8)
**Location of thrombus**	
Common femoral vein	29 (41.4)
External iliac vein	17 (24.3)
Common iliac vein	14 (20)
Distal femoral vein	7 (10)
Internal iliac vein	1 (1.4)
Other vein	2 (2.9)
**Illness severity**	
qSOFA score ≥2	7 (10)

SIRS criteria ≥2	46 (65.7)
ICU admission	13 (18.6)
Vasopressors	12 (17.1)
Invasive mechanical ventilation	5 (7.1)
Renal replacement therapy	1 (1.4)

Data shown as n(%) unless otherwise stated.

[a]Denominator = 70 unless otherwise stated

IQR: interquartile range; HIV: human immunodeficiency virus; HBV: hepatitis B virus; HCV: hepatitis C virus; BP: blood pressure; SpO_2: oxygen saturations; qSOFA: quick sequential organ failure assessment; SIRS: systemic inflammatory response syndrome; ICU: intensive care unit.

Supplementary Table 2: Microbiological findings

Bacteria	Total	Blood	Intra-operative
Staphylococci			
Staphylococcus aureus	33	24	9
Coagulase-negative	5	2[a]	3
Streptococci			
Anginosus group	10	4	6
Viridans group[b]	5	5	0
Streptococcus pyogenes	5	4	1
Streptococcus dysgalactiae	3	2	1
Anaerobes			
Actinomyces sp.	4	4	0
Fusobacterium sp.	2[c]	1	1
Other/Not specified	5	3	2
Gram negative			
Escherichia coli	3[c]	1	2
Other gram positive			
Enterococcus faecalis	1[c]	1	0
Helcococcus kunzii	1[c]	1	0
Lactobacillus fermentum	1[c]	0	1
Trueperella bernardiae	1[c]	1	0

[a] both patients with coagulase-negative Staphylococci isolated from blood culture also had other pathogens identified in blood cultures (n=1 *Streptococcus anginosus*; n=1 *Actinomyces turicensis* plus *Fusobacterium gonidiaformans*).
[b] excluding Anginosus group Streptococci
[c] identified exclusively from polymicrobial infections

Supplementary Table 3: Classification of antimicrobial spectra

Spectrum	Included antimicrobials
Gram positive including *S. aureus*	Flucloxacillin, vancomycin, clindamycin, piperacillin-tazobactam, ceftriaxone, amoxicillin-clavulanic acid, meropenem
Gram positive not including *S. aureus*	Penicillin, amoxicillin
Gram negative	Gentamicin, ciprofloxacin, piperacillin-tazobactam, ceftriaxone, amoxicillin-clavulanic acid, meropenem
Anaerobic	Metronidazole

Supplementary Table 4: Cumulative antimicrobial usage

Group	Included antimicrobials	N days	% total days
Anti-staphylococcal gram positive	Flucloxacillin, vancomycin, daptomycin, linezolid, clindamycin, doxycycline	1827	**42.2**
Anaerobic	Metronidazole	1078	**24.9**
Broad-spectrum	Piperacillin-tazobactam, amoxicillin-clavulanic acid, ceftriaxone, meropenem	561	**13.0**
Narrow-spectrum gram positive	Penicillin, amoxicillin	430	**9.9**
Gram negative	Gentamicin, ciprofloxacin	380	**8.8**
Other	Clarithromycin, rifampicin	27	**0.6**
Unknown		19	**0.4**
Anidulafungin		3	**0.1**

Supplementary Table 5: Univariable analyses of factors associated with clinical failure

Variable	Measure	Cure	Failure	p-value[c]
Bacteraemia	N	37/58	2/8	0.055
S. aureus	N	26/62	3/8	1.0
Septic shock[a]	N	12/62	0/8	0.3
qSOFA ≥2[b]	N	6/55	1/7	1.0
SIRS ≥2[b]	N	43/59	3/8	0.1
Total WCC (x10^9/L)[b]	Median	12.8	11.5	0.6
NLR[b]	Median	8.1	8.6	0.6
CRP (mg/L)[b]	Mean	229.4	238.4	0.8
Peak CRP (mg/L)	Mean	256.5	248.5	0.8
Metastatic infection	N	26/62	3/8	1.0
Local abscess	N	22/62	2/8	0.7
Initial gram-negative coverage	N	44/61	4/8	0.2

[a] requirement for vasopressors
[b] measured at admission
[c] un-adjusted p-value
WCC: white cell count; NLR: neutrophil:lymphocyte ratio; CRP: C-reactive protein

Supplementary Table 6: Findings from previous observational studies

	**Hakeem & Bhattacharyya** [1]	**Fäh *et al*** [2]	**Mertz *et al*** [3]
Location	U.K.	Switzerland	Switzerland
Design	Retrospective cohort	Retrospective cohort	Retrospective cohort
Denominator	10 (patients)	7 (patients)	36 (episodes)
Age, years	34 (mean)	30 (median)	33 (median)
Male:Female	8:2	3:4	22:14
DVT location	Ileofemoral (10)	Ileofemoral (5)<br>Internal jugular (1)<br>Upper arm (1)	Ileofemoral (28)<br>Internal jugular (5)<br>Saphenous (2)<br>Subclavian (1)
Complications			
Septic PE	5	2	5
IE	1	0	3
Microbiology[a]			
S. aureus	5	4	19
Streptococci	4	2	14
Gram negative	0	0	0
HCV	4	3	4
Total antimicrobial duration (d), median (range)	29.5 (15-64)	28 (10-41)	19.5 (6-85)
Clinical cure[b]	8/10	5/7	34/36

[a]Blood cultures only
[b]Defined as relapse of same infection within one year

References

1. Hakeem MBD. Septic deep venous thrombophlebitis and distant emboli in injecting drug users – treatment experience and outcome. J R Coll Physicians Edinb **2007**; 37(4): 293-9.
2. Fäh F, Zimmerli W, Jordi M, Schoenenberger RA. Septic deep venous thrombosis in intravenous drug users. Swiss Med Wkly **2002**; 132(27-28): 386-92.
3. Mertz D, Khanlari B, Viktorin N, Battegay M, Fluckiger U. Less than 28 days of intravenous antibiotic treatment is sufficient for suppurative thrombophlebitis in injection drug users. Clin Infect Dis **2008**; 46(5): 741-4.
